# Cuffless and Continuous Blood Pressure Estimation from the Heart Sound Signals

**DOI:** 10.3390/s150923653

**Published:** 2015-09-17

**Authors:** Rong-Chao Peng, Wen-Rong Yan, Ning-Ling Zhang, Wan-Hua Lin, Xiao-Lin Zhou, Yuan-Ting Zhang

**Affiliations:** 1 Shenzhen Institutes of Advanced Technology, Chinese Academy of Sciences, Shenzhen 518055, China; E-Mails: pengxiaotu@126.com (R.-C.P.); nl.zhang@siat.ac.cn (N.-L.Z.); wh.lin@siat.ac.cn (W.-H.L.); ytzhang@ee.cuhk.edu.hk (Y.-T.Z.); 2 Shenzhen College of Advanced Technology, University of Chinese Academy of Sciences, Shenzhen 518055, China; 3 Key Lab for Health Informatics of Chinese Academy of Sciences (HICAS), Shenzhen 518055, China; 4 Department of Physics and Materials Science, City University of Hong Kong, Hong Kong 999077, China; E-Mail: christopheryanwr@gmail.com; 5 Department of Electronic Engineering, Chinese University of Hong Kong, Hong Kong 999077, China

**Keywords:** blood pressure, cross-validation, heart sound, smartphone, support vector machine

## Abstract

Cardiovascular disease, like hypertension, is one of the top killers of human life and early detection of cardiovascular disease is of great importance. However, traditional medical devices are often bulky and expensive, and unsuitable for home healthcare. In this paper, we proposed an easy and inexpensive technique to estimate continuous blood pressure from the heart sound signals acquired by the microphone of a smartphone. A cold-pressor experiment was performed in 32 healthy subjects, with a smartphone to acquire heart sound signals and with a commercial device to measure continuous blood pressure. The Fourier spectrum of the second heart sound and the blood pressure were regressed using a support vector machine, and the accuracy of the regression was evaluated using 10-fold cross-validation. Statistical analysis showed that the mean correlation coefficients between the predicted values from the regression model and the measured values from the commercial device were 0.707, 0.712, and 0.748 for systolic, diastolic, and mean blood pressure, respectively, and that the mean errors were less than 5 mmHg, with standard deviations less than 8 mmHg. These results suggest that this technique is of potential use for cuffless and continuous blood pressure monitoring and it has promising application in home healthcare services.

## 1. Introduction

Cardiovascular disease (e.g., hypertension, arteriosclerosis, coronary heart disease) is one of the top killers of human life. As reported by the World Health Organization (WHO), it took 17.5 million people’s lives globally in 2012 [1]. The number will increase as the population ages. Therefore, early detection and prevention of the cardiovascular disease is of significant importance to promote people’s health. However, traditional medical devices used in the hospital are often bulky and expensive, only operated by specially trained nurses, and not suitable for home healthcare. Hence, there is a need of portable, low-cost devices that can be easily operated by ordinary people to detect physiological parameters (e.g., heart rate, breath rate, blood pressure) for self-monitoring at home.

As the smartphone is becoming ubiquitous, its application in medicine is of increasing interest [2]. The new generation smartphones have larger memories, more powerful CPUs, and more built-in sensors to collect data from the outside world, making it possible to detect physiological parameters using a smartphone without the help of external sensors. Many researchers have demonstrated that the smartphone can be used to detect heart rate [3,4,5,6,7], respiratory rate [5], pulse volume [7], and oxygen saturation [5]. These new techniques require no specialized hardware but only software installed in the smartphone, so that they can be used anywhere, anytime, by anyone, and have great potential to be used in home healthcare services in the future.

In this paper, we demonstrate that the smartphone can also be used to estimate blood pressure. Blood pressure is the pressure exerted on the wall of blood vessels by blood when the blood flows through arterial vessels [8]. It is an essential parameter for the diagnosis and treatment of cardiovascular disease, and daily monitoring of blood pressure is also important for the prevention of cardiovascular disease among normal people. To the best of our knowledge, the most common method to measure blood pressure is to use a mercury sphygmomanometer with an inflatable cuff; however, it is rarely reported in previous literature to measure blood pressure with a smartphone. Lamonaca *et al.* applied an artificial neural network to evaluate blood pressure from the pulse wave signal acquired by the camera of the smartphone [9,10]. Chandrasekaran *et al.* proposed two other methods to cufflessly estimate blood pressure with smartphones [11]. The first method used two smartphones to separately acquire the heart sound and the pulse wave signals, and then calculated vascular transit time for blood pressure estimation. The second method used a single smartphone to acquire the pulse wave signal and a custom external microphone to acquire the heart sound, and also calculated the vascular transit time. Different from these above, a novel method was employed herein to estimate continuous blood pressure from the heart sound signal acquired by a smartphone. It is based on the principle that the pattern of the second heart sound (S2) is associated with the blood pressure [12]. If the pattern of the S2 is recognized, then the blood pressure will be determined.

## 2. Methods

### 2.1. Dependence of S2 upon Blood Pressure

Heart sounds are mixed audible sounds generated by the contraction and relaxation of the atria and ventricles, the valve movements, and the blood flow [13]. The two primary components of the heart sounds in a heart cycle are the first heart sound (S1) and the second heart sound (S2). S1 is produced by nearly simultaneous closure of the mitral and the tricuspid valve, and S2 is produced by nearly simultaneous closure of the aortic and the pulmonic valve [14].

It is well-recognized in clinical medicine that the S2 has a characteristic “accentuation” in hypertensive patients [12,15]. Bartels *et al.*, explained this phenomenon as the mechanical oscillation caused by the elasticity of the vessel walls and the inertia of the blood column. When the blood pressure is increasing, the arterial wall will exert an increasing reset force to counteract the tangential tension of the arterial wall, resulting in increasing oscillation frequencies of the blood column [12]. According to this, Zhang *et al.*, built a mathematical model for the vibration of the closed aortic valve, and the simulation results showed that the increasing aortic pressure lead to an increase both in frequency and amplitude of the produced sound [16]. Later, Bombardini *et al.* utilized a force sensor to collect heart sound signals and carefully investigated the relationship between S2 and blood pressure in 146 patients. They found that the S2 recordings quantitatively documented the blood pressure changes and that the correlation coefficients between the amplitude of S2 and systolic, diastolic, and mean blood pressure were 0.544, 0.502, and 0.567, respectively [17]. Accordingly, it is possible to non-invasively estimate blood pressure from the heart sound signals. We herein developed a regression model between the Fourier spectrum of S2 and blood pressure using a support vector machine, and then used this model to estimate blood pressure from the heart sound signals acquired by a smartphone.

### 2.2. Data Acquisition

The experiment was approved by the Institutional Review Board of Shenzhen Institutes of Advanced Technology (registration number: SIAT-IRB-120515-H0009). Thirty-two subjects participated in the experiment (25 males and 7 females, age 20–32 years, height 150–185 cm, weight 44–90 kg). They were all healthy without any known diseases and provided their written informed consent. They were asked to refrain from caffeine, alcohol, cigarettes or strenuous exercise for 2 h before the experiment.

**Figure 1:**
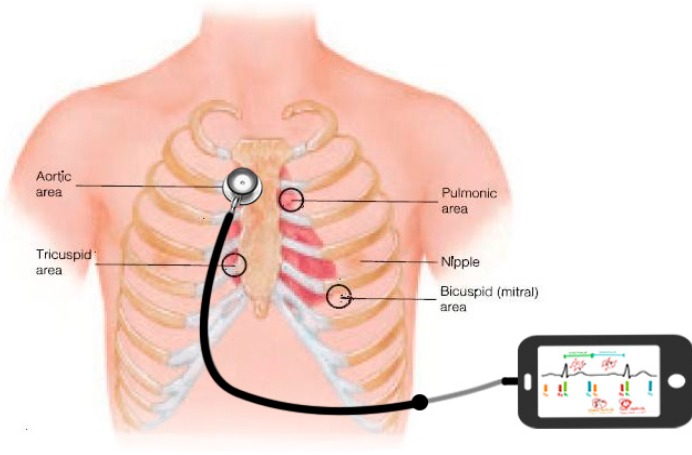
Schematic diagram for heart sound acquisition using a smartphone. The stethoscope is used to enhance the acoustic wave and the microphone on the earphone line is fixed in the hollow tube of the stethoscope.

In the experiment, all the subjects were instructed to lie in the supine position on a mattress. As shown in Figure 1, a smartphone was used to collect the heart sound signals. The microphone on the earphone line was fixed in the hollow tube of a stethoscope, and the stethoscope was placed on the chest of the subject to enhance the acoustic wave. Since we were interested in the S2, the stethoscope was located at the right upper sternal border where the S2 is stronger than that in other regions. Simultaneously, a Finometer^®^ MIDI (Model II, Finapres Medical Systems B.V., Amsterdam, The Netherlands) was used with a finger cuff around the right middle finger to measure continuous blood pressure. The Finometer is a commercial medical device that can measure beat-to-beat blood pressure based on the volume-clamping principle. It provided a report of the systolic, diastolic and mean blood pressure for each heartbeat.

**Figure 2:**
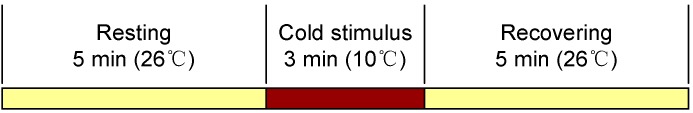
The procedure of the cold pressor test.

As shown in Figure 2, the experimental procedure lasted 13 min for each subject, including three stages. Firstly, the subject had a rest lying on the mattress for 5 min, with the room temperature conditioned at 26 °C. Secondly, the subject was instructed to immerse his/her left hand into the 10 °C cold water and to keep in the water for 3 min, so that the sympathetic nerves were evoked to elevate the blood pressure. Thirdly, the subject took his/her hand out of the water and had another rest for 5 min. During the whole experimental process, the subject was asked not to speak and to keep as still as possible to reduce random noise and motion artifact.

### 2.3. Identification of S2

All the data were processed offline in Matlab 7.0 (The Mathworks Inc., Natick, MA, USA) on a personal computer. The heart sound signals were first filtered by a Butterworth low-pass filter with cutoff frequency of 1000 Hz to reduce high-frequency noise, and then were filtered by a Butterworth high-pass filter with cutoff frequency of 5 Hz to remove the baseline wandering. These filters were implemented by filtering the signal in both forward and backward direction to achieve a zero-phase response without group delay. As the heart sound signals were sampled at 44.1 kHz by the smartphone, they were decimated by a factor of 20 to obtain a lower sampling frequency of 2205 Hz.

Then each down-sampled signal was segmented based on its envelope of Shannon energy [18]. Firstly, the signal was normalized to the range of [−1, 1] by dividing by its absolute maximum. Secondly, the Shannon energy was calculated according to the equation as:

(1) $$E=-x^{2}\log(x^{2})$$

where *x* is the normalized signal. Thirdly, the Shannon energy was averaged in a moving time window of 20 ms with overlap of 10 ms, as expressed by:

(2) $$E_{A}=\frac{1}{N}\sum_{i=1}^{N}E$$

where *E* is the Shannon energy, *E_A_* is the average Shannon energy and *N* is the length of the window. Since the sampling frequency was 2205 Hz, *N* was equal to 44 here. Then, *E_A_* was normalized by subtracting the mean and dividing by the standard deviation, as expressed by:

(3) $$E_{N}=\frac{E_{A}-M(E_{A})}{S(E_{A})}$$

where *E_N_* is the normalized average Shannon energy, M(*E_A_*) and S(*E_A_*) are its mean value and standard deviation, respectively.

**Figure 3:**
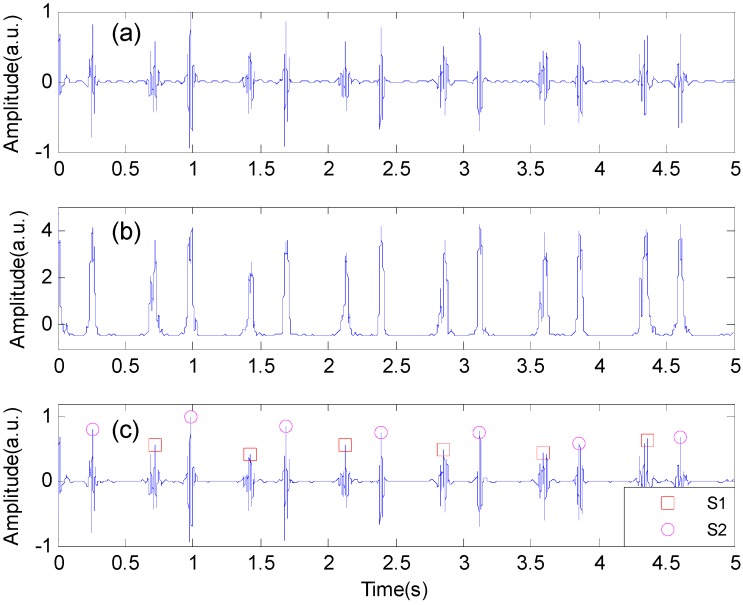
Identification of the second heart sound using the Shannon energy envelope. (**a**) The normalized heart sound signal; (**b**) The normalized average Shannon energy; (**c**) The first heart sounds and the second heart sounds marked with squares and circles, respectively.

Afterwards, two thresholds were applied to *E_N_* to identify the potential peaks of S1 and S2 [18]. A high threshold was set as a preset coefficient (usually 0.2–0.4) times the mean value of the five largest amplitudes, in order to detect the large-amplitude peaks and to eliminate the effect of noise. A low threshold was set as half of the mean value of the envelope. It was slightly higher than the background noise and was used to pick up the low-amplitude peaks that might be regarded as noise by the high threshold. These peaks were then classified as S1 or S2 according to the clinical knowledge that the duration from S1 to S2 is shorter than that from S2 to S1. An example of the identification process of S1 and S2 are shown in Figure 3. In some cases that the heart sound signals were disturbed and the S1 and S2 were not correctly identified, for example when the subject spoke or cough, the S1 and S2 were manually removed or adjusted by visual inspection on the computer screen.

### 2.4. Regression Using Support Vector Machine

**Figure 4:**
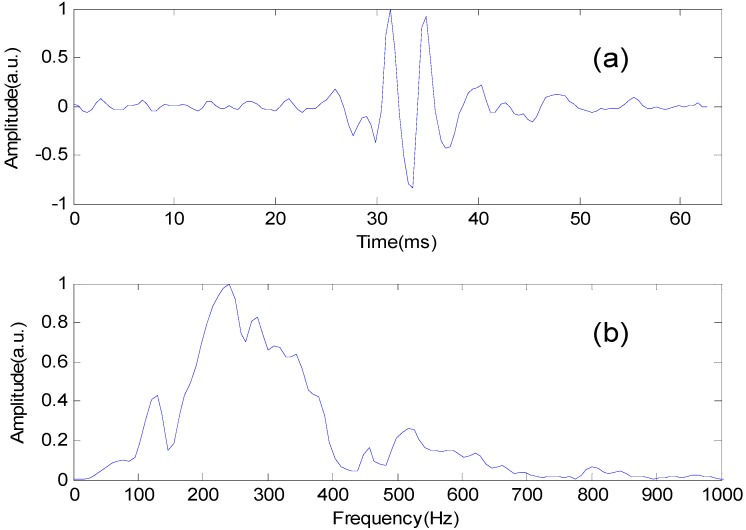
The second heart sound and its frequency spectrum.

Once identified, the S2 of each heartbeat in the normalized heart sound signals was truncated by a 64 ms window centered at the maximum of the S2, and its frequency spectrum was obtained by fast Fourier transform (FFT), shown in Figure 4. The spectrum was then normalized by dividing its maximum value, and the 36 spectral values with 10 Hz interval in the frequency band 50–400 Hz were chosen as the S2 features.

Afterwards, the S2 features were separately connected with the systolic, diastolic and mean blood pressure using a support vector machine (SVM). The SVM is a popular machine learning method for classification and regression [19]. The regression of the SVM is to solve the convex optimization problem defined as below [20]:

(4) $$\begin{array}{l} \mathrm{minimize}\frac{1}{2}∥w{∥}^{2}\\ \begin{array}{cc} \mathrm{subject} & \begin{array}{ccc} \mathrm{to} & \left\{\begin{array}{c} y_{i}-\langle w,x_{i}\rangle -b\leq \varepsilon \\ \langle w,x_{i}\rangle +b-y_{i}\leq \varepsilon \end{array}\right\} & \end{array} \end{array} \end{array}$$

where *x_i_* is a feature vector with target value *y_i_*, *w* is weight vector, *b* is intercept, $\langle w,x_{i}\rangle +b$ is the prediction value for the feature vector *x_i_*, and *ε* is a threshold of the deviation. The SVM regression maps the feature vectors *x_i_* (e.g., the S2 features) into a higher dimensional space by a nonlinear transform, and finds an optimal linear hyperplane with at most *ε* deviation to fit the target value *y_i_* (e.g., the blood pressure) in this higher dimensional space [21]. The nonlinear transform is usually implemented by properly defining an inner product function, also called the kernel function, such as polynomial function, radial basis function (RBF), and sigmoid function. Here the RBF kernel was chosen for the regression of the S2 features and the blood pressure, because it is widely used in many applications and generally has a good performance.

The LIBSVM package developed by Chih-Chung Chang and Chih-Jen Lin [19] was employed for the SVM training and testing. As there were no testing data, the 10-fold cross-validation[22] was used to test the accuracy of the regression model. For each subject, the sample data (Feature vector: the S2 features. Target value: the systolic, diastolic, and mean blood pressure) were divided into 10 subsets of equal size. Nine subsets were used for training the regression model and the other one was retained for testing the accuracy of the model. Then another nine subsets were used for training and the remaining one for testing, and so on. This cross-validation process was repeated 10 times so that each instance in the whole dataset was tested exactly once.

### 2.5. Statistical Analysis

For each subject, the predicted values using the SVM regression were compared with the ‘true’ values measured by the Finometer device. Pearson correlation coefficient (CC), mean absolute error (MAE), mean error (ME), and standard deviation (SD) were calculated as below:

(5) $$\mathrm{CC}=\frac{\sum_{i=1}^{n}(x_{i}-\bar{x})(y_{i}-\bar{y})}{\sqrt{\sum_{i=1}^{n}{\left(x_{i}-\bar{x}\right)}^{2}}\sqrt{\sum_{i=1}^{n}{\left(y_{i}-\bar{y}\right)}^{2}}}$$

(6) $$\mathrm{MAE}=\frac{1}{n}\sum_{i=1}^{n}\left|y_{i}-x_{i}\right|$$

(7) $$\mathrm{AE}=\frac{1}{n}\sum_{i=1}^{n}\left(y_{i}-x_{i}\right)$$

(8) $$\mathrm{SD}=\sqrt{\frac{1}{n-1}\sum_{i=1}^{n}{\left(y_{i}-x_{i}-ME\right)}^{2}}$$

where *y* is the predicted value, *x* is the measured value, and *n* is the number of samples.

## 3. Results

**Figure 5:**
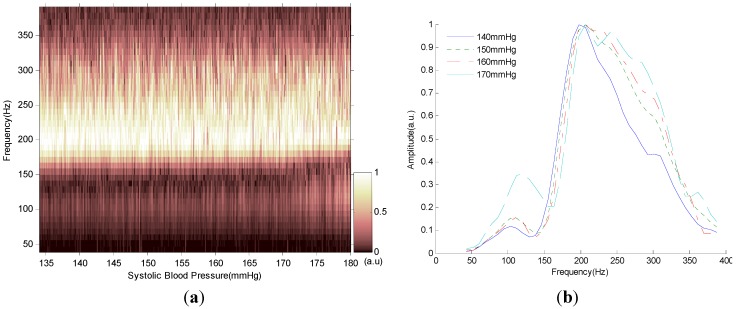
Spectral change of the second heart sound with blood pressure variation. (**a**) Normalized Fourier spectrum of the second heart sound with systolic blood pressure increasing. (**b**) Normalized Fourier spectrum of the second heart sound when the systolic blood pressure was 140, 150, 160 and 170 mmHg.

The relationship between the spectrum of the S2 and blood pressure is shown in Figure 5. We can see in Figure 5a that when the blood pressure was increasing, the normalized Fourier spectrum of the S2 was slightly shifting upward to the higher frequency. This phenomenon was more clearly shown in Figure 5b that when the blood pressure increased from 140 to 170 mmHg, the Fourier spectrum of the S2 shifted rightward to the higher frequency and the spectral peak became wider. We can thus infer that it was possible to predict blood pressure from the Fourier spectrum of the S2.

**Figure 6:**
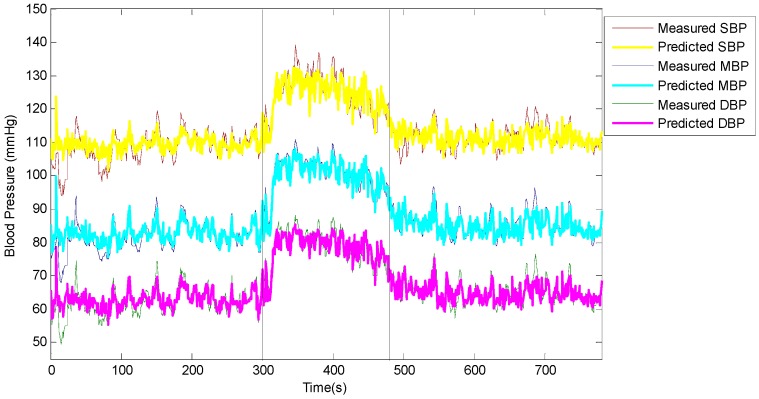
Variation of predicted values and measured values for systolic blood pressure (SBP), diastolic blood pressure (DBP) and mean blood pressure (MBP) during the cold pressor test. The vertical lines indicate the beginning and the end of the cold stimulus.

**Figure 7:**
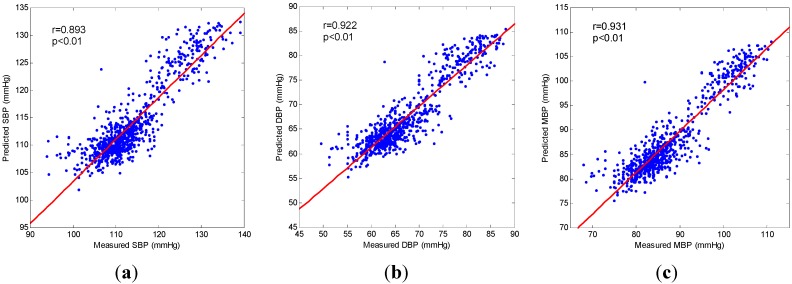
Correlation analysis between predicted values and measured values for (**a**) Systolic blood pressure (SBP); (**b**) Diastolic blood pressure (DBP); (**c**) Mean blood pressure (MBP). In each plot, the diagonal is the linear regression line; r, Pearson correlation coefficient.

A typical example of blood pressure estimation was given in Figure 6 and Figure 7.The predicted values of the systolic, diastolic, and mean blood pressure using the SVM regression were compared with the corresponding values measured by the Finometer device. It is clearly shown in Figure 6 that the predicted values increased as the measured values increased at the beginning of the cold stimulus, and that the predicted values decreased as the measured values decreased after the cold stimulus. Namely, they had a close correlation. In Figure 7 the correlation analysis shows that the CCs between the predicted values and the measured values were 0.893, 0.922, and 0.931 for systolic, diastolic and mean blood pressure, respectively. It is apparent that the dots in each plot clustered around the linear regression line and distributed equally on both sides, which indicated that the predicted values were closely correlated to the measured values.

**Figure 8:**
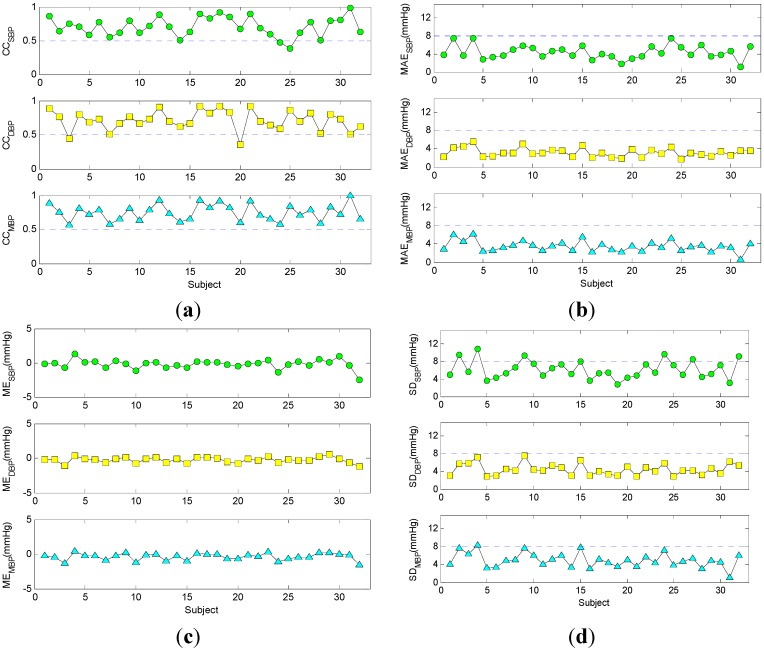
Comparison of predicted values and measured values for each subject. (**a**) Correlation coefficients (CC); The presented data were all statistically significant (*p* < 0.05); (**b**) Mean absolute error (MAE); (**c**) Mean error (ME); (**d**) Standard deviation (SD). SBP, systolic blood pressure; DBP, diastolic blood pressure; MBP, mean blood pressure.

The CCs, MAEs, MEs, and SDs for each subject were presented in Figure 8. As shown in Figure 8a, most of the CCs were greater than 0.5. This meant that the predicted values and measured values were strongly correlated in most cases. In Figure 8b, all the MAEs were less than 8 mmHg. This meant that the predicted values were very close to the measured values with their distance less than 8 mmHg. In Figure 8c, the MEs were approximately to zero and had a small fluctuation up and down. This meant that the predicted values were nearly unbiased to the measured values and there was only a small systematic error between them. In Figure 8d, most of the SDs were less than 8 mmHg, except for the systolic blood pressure in a few subjects. This meant that the variations of the difference between the predicted values and measured values were small enough.

The distributions of CC, MAE, ME, and SD among all the subjects were presented in Table 1. The mean CCs were 0.707, 0.712 and 0.748 for systolic, diastolic, and mean blood pressure, respectively. The mean MEs were −0.204, −0.274, and −0.357 mmHg for systolic, diastolic, and mean blood pressure, respectively. The mean SDs were 6.121, 4.471, and 4.961 mmHg for systolic, diastolic, and mean blood pressure, respectively. These results were better than those from the pulse transit time (PTT) model which is commonly used in literature for blood pressure estimation, as we have done some preliminary work on the PTT model and the mean CCs for PTT *vs.* SBP and PTT *vs.* DBP were −0.544 and −0.451, respectively. Additionally, as required by the American Association for the Advancement of Medical Instrumentation (AAMI), the ME should be ± 5 mmHg or less, with a SD of 8 mmHg or less [23]. These results suggested that this technique might provide effective estimates of blood pressure with sufficient accuracy.

**Table 1:** Distributions of the statistical parameters among all the subjects.

Parameter	Maximum	Median	Minimum	Mean
CC_SBP_	0.981	0.707	0.386	0.707
CC_DBP_	0.923	0.716	0.358	0.712
CC_MBP_	0.996	0.742	0.567	0.748
MAE_SBP_(mmHg)	7.472	3.846	1.050	4.339
MAE_DBP_(mmHg)	5.472	3.040	1.767	3.171
MAE_MBP_(mmHg)	6.101	3.459	0.585	3.480
ME_SBP_(mmHg)	1.231	−0.108	−2.494	−0.204
ME_DBP_(mmHg)	0.496	−0.174	−1.190	−0.274
ME_MBP_(mmHg)	0.463	−0.247	−1.490	−0.357
SD_SBP_(mmHg)	10.708	5.452	2.815	6.121
SD_DBP_(mmHg)	7.488	4.225	2.878	4.471
SD_MBP_(mmHg)	8.383	4.819	1.014	4.961

CC, Pearson correlation coefficient; MAE, mean absolute error; ME, mean error; SD, standard deviation; SBP, systolic blood pressure; DBP, diastolic blood pressure; MBP, mean blood pressure.

## 4. Discussion

The results confirmed that the spectrum of S2 had a strong connection with the blood pressure, and that it was realizable to estimate blood pressure from the heart sound acquired by a smartphone. This new technique can continuously and non-invasively measure beat-to-beat blood pressure using only a smartphone and a stethoscope. It needs neither the sphygmomanometer with an inflatable cuff, nor the bulky and expensive medical instrument used only in the hospital. It is easy-to-use and low in cost, and well-suited for daily use at home. As we have tested, when the environment is quiet, even the stethoscope is not required and the heart sound can be clearly detected by just pressing the microphone tightly on the chest of the subject [11], or using a plastic funnel instead of the conventional stethoscope to enhance the acoustic wave. Furthermore, this technique can be extensively applied to wearable medical devices. For example, the microphone can be minimized into a small piece, pasted on the chest of the subject to acquire the heart sound, and wirelessly communicate with a smartphone or a computer so that the blood pressure can be monitored all day without any disturbance to the user’s daily life. In short, the technique of estimating blood pressure from heart sound has great potential in home healthcare applications.

Although the results are encouraging, there are some technical issues which need to be taken into account to use this technique in practical situations. The first is to reduce the training phase. In the presented experiment, to collect the training data, we elevated the blood pressure by the cold stimulus, which cannot be applied to cardiovascular patients due to its potential risk of strokes or heart attacks. Therefore, the wide blood pressure variation needs to be simplified to a safe and convenient procedure, for example, collecting the training data before and after physical exercise [24], or changing the blood pressure by raising the hand at different heights [25,26]. Another possible way to reduce the training phase is to build a general mathematical model for all people or a family of models for specific age and gender groups [27]. If the model or models are well-trained by massive clinical datasets and served as the calibrated benchmark for continuous blood pressure monitoring, then there is no need for personalized calibration for each individual.

The second is to implement a real-time SVM algorithm on the smartphone. At the current stage, the SVM algorithm runs on a personal computer and all the signals are processed offline. In order to monitor blood pressure in real-time, it is important to port the SVM algorithm to a smartphone platform where the CPU power and memories are limited. However, as the smartphone technology is rapidly developing, we think this is not a huge problem.

The third is to reduce the interference of environmental noise. The heart sound is so weak that it may be corrupted by the environmental noise. We suggest strengthening the heart sound signals in two aspects. In the hardware aspect, the microphone can be designed as a small piece pasted on the chest of the subject, with a small funnel embedded inside to collect acoustic wave, and with sponges covered outside to prevent environmental noise. However, this design can be only applied to home-made wearable devices, and is not applicable to off-the-shelf smartphones. In the software aspect, adaptive filters [28] can be used to eliminate the external noise, and robust segmentation algorithms [29,30] can be used to improve the performance of S2 detection in a noisy environment.

## 5. Conclusions

In this paper, with a smartphone to collect heart sound signals, we demonstrate a new technique to estimate blood pressure from the spectra of the second heart sound, continuously, non-invasively and without a cuff. This technique provides an easy, low-cost and comfortable solution for daily monitoring of blood pressure, which is especially useful for cardiovascular patients.
